# Direct Analysis of Solid-Phase Carbohydrate Polymers by Infrared Multiphoton Dissociation Reaction Combined with Synchrotron Radiation Infrared Microscopy and Electrospray Ionization Mass Spectrometry

**DOI:** 10.3390/polym17172273

**Published:** 2025-08-22

**Authors:** Takayasu Kawasaki, Heishun Zen, Kyoko Nogami, Ken Hayakawa, Takeshi Sakai, Yasushi Hayakawa

**Affiliations:** 1 Accelerator Laboratory, High Energy Accelerator Research Organization, 1-1 Oho, Tsukuba 305-0801, Ibaraki, Japan; 2 Institute of Advanced Energy, Kyoto University, Gokasho, Uji 611-0011, Kyoto, Japan; zen@iae.kyoto-u.ac.jp; 3 Laboratory for Electron Beam Research and Application (LEBRA), Institute of Quantum Science, Nihon University, 7-24-1 Narashinodai, Funabashi 274-8501, Chiba, Japan; nogami.kyoko@nihon-u.ac.jp (K.N.); hayakawa.ken@nihon-u.ac.jp (K.H.); sakai.takeshi@nihon-u.ac.jp (T.S.); hayakawa.yasushi@nihon-u.ac.jp (Y.H.)

**Keywords:** infrared, free electron laser, carbohydrates, polysaccharides, vibrational excitation, multiphoton dissociation, synchrotron radiation

## Abstract

To determine the structure of carbohydrate polymers using conventional analytical technology, several complicated steps are required. We instead adopted a direct approach without the need for pretreatments, using an intense infrared (IR) laser for carbohydrate analysis. IR free-electron lasers (FELs) driven by a linear accelerator possess unique spectroscopic features, including extensive wavelength tunability and high laser energy in the IR region from 1000 cm^−1^ (10 μm) to 4000 cm^−1^ (2.5 μm). FELs can induce IR multiphoton dissociation reactions against various molecules by supplying vibrational excitation energy to the corresponding chemical bonds. Chitin from crayfish and cellulose fiber were irradiated by FELs tuned to νC–O (9.1–9.8 μm), νC–H (3.5 μm), and δH–C–O (7.2 μm) in glycosidic bonds, and their low-molecular-weight sugars were separated, which were revealed by combining synchrotron radiation IR spectroscopy and electrospray ionization mass spectrometry. An intense IR laser can be viewed as a “molecular scalpel” for dissecting and directly analyzing the internal components in rigid biopolymers. This method is simple and rapid compared with general analytical techniques.

## 1. Introduction

Along with proteins, nucleic acids, and lipids, carbohydrates, which are polymers, are key components in biochemical systems [1,2,3]. Polysaccharides (containing more than 20 monomeric sugars) exist in various states in nature, such as components of plant tissues [4,5], cellular matrices of animal tissues [6,7], outer shells of insects and crustaceans [8,9,10], and cell walls of microorganisms [11,12]. Based on their chemical composition, two types of natural polysaccharides exist: homoglycans and heteroglycans. In the former type, amylose, amylopectin, and glycogen are composed of glucose units that are linked via α-1,4 or α-1,6 bonds [13,14,15]. Cellulose is polymerized by β-1,4 bonds [16,17], and chitin is constructed by β-1,4GcNAc units [18,19]. Both polysaccharides are structured by fiber-like carbohydrate chains, in contrast to the globular chains of starch. Heparin is composed of glucosamine and iduronic acid [20,21], hyaluronic acid is composed of glucuronic acid and *N*-acetylglucosamine [22,23], and glucomannan is polymerized by glucose and mannose [24,25]. These carbohydrate polymers play important roles in the expression of biological functions and are potential resources for pharmaceutical agents and food additives [26,27]. To understand the functional roles of these carbohydrates in biological systems, determining the detailed structures of polysaccharides is necessary.

In many cases, characterizing oligomers or monomers is important for analyzing the entire structure of the parent carbohydrate polymers. Conventionally, colorimetric assays are used [28,29,30,31,32,33]. For example, anthrone–sulfuric acid can be widely applied to aldose and reducing sugars [28,29,30], the Elson–Morgan method using the Ehrlich reagent is used to detect glucosamine [31], and carbazole–sulfuric acid is effective for the detection of uronic acids [32,33]. In clinical chemistry, a fluorescence-labeling method has been developed to detect small amounts of sugars in biological tissues [34,35]. Blood glucose has been quantified using fluorescently labeled boronic acid derivatives [34], and glucose and lactose in serum have been detected using a fluorescent iron complex combined with oxidases [35]. To analyze oligosaccharide structures, various fluorescent-labeling reagents have been developed, and particularly, 2-amino pyridine is useful for derivatizing oligosaccharides for detection using high-performance liquid chromatography (HPLC) [36,37]. Capillary electrophoresis (CE) can also be a powerful tool for the more sensitive investigation of the molecular weights of various types of sugars than conventional HPLC [38,39].

The major analytical techniques used for the structural analysis of natural polysaccharides are listed in Table 1.

Amylose content is analyzed using various quantitative and qualitative methods, including near-infrared (NIR) and Raman spectroscopy, CE, and atomic force microscopy (AFM) [40]. In addition, size exclusion chromatography (SEC), multiple-angle laser light scattering (MALLS), and ^1^H nuclear magnetic resonance (NMR) were employed to analyze the soluble fraction of rice flour in hot water [41]. Amylopectin from cornstarch was analyzed using SEC, MALLS, and differential scanning calorimetry (DSC) [42]. In addition, starch alteration by enzymatic hydrolysis has been thoroughly investigated using scanning electron microscopy (SEM), X-ray diffraction (XRD), Fourier transform infrared (FT-IR) spectroscopy, ^1^H NMR spectroscopy, high-performance anion exchange chromatography with pulsed amperometry detection (HPAEC-PAD), DSC, and rapid viscosity analysis (RVA) [43]. In particular, HPAEC-PAD is a reliable tool for determining the carbohydrate chain-length distribution in natural polysaccharides [44]. Glycogens associated with lung disease can be analyzed using matrix-assisted laser desorption/ionization mass spectrometry imaging (MALDI-MSI) combined with *N*-glycosidase hydrolysis [45]. Photoacoustic spectroscopy (PAS) and ^1^H NMR spectroscopy can be utilized to investigate the fiber and crystalline conformations of cellulose [46]. In addition, thermogravimetry (TG) was introduced as a new method for determining the relative contents of cellulose, hemicellulose, and lignin in biomass samples [47]. Chitin can be analyzed using various methods, including FT-IR spectroscopy, XRD, SEM, UV–Vis spectroscopy, mass spectrometry (MS), and NMR spectroscopy [48,49]. Heparin can be quantitatively analyzed by enzymatic digestion followed by liquid chromatography (LC)–MS and strong anion exchange (SAX) chromatography [50]. In particular, attenuated total reflectance (ATR) FT-IR was employed to analyze solid-phase samples from a library of various glycosaminoglycans [51]. The molecular distribution of hyaluronic acid has been analyzed using an HPLC-SEC system [52], and a solid-state nanopore sensor has been developed as a more sensitive detector [53]. The sugar composition of glucomannan can be analyzed using ion exchange chromatography and gel permeation chromatography, followed by thin-layer chromatography (TLC), FT-IR spectroscopy, and ^13^C NMR spectroscopy [54]. *N*-glycan is analyzed using CE combined with MALDI-MS and electrospray ionization (ESI)-MS [55].

**Table 1:** Various analytical techniques for natural carbohydrate polymers.

Carbohydrate Polymers	Analytical Techniques	Ref.
Amylose	NIR, Raman, CE, AFM SEC, MALLS, ^1^H NMR	[40] [41]
Amylopectin	SEC, MALLS, DSC SEM, XRD, FT-IR, ^1^H NMR, HPAEC-PAD, DSC, RVA	[42] [43]
Glycogen	MALDI-MSI, *N*-glycosidase hydrolysis	[45]
Cellulose	^1^H NMR, PAS TG	[46] [47]
Chitin	FT-IR, XRD, SEM XRD, FT-IR, UV-Vis, MS, ^13^C NMR	[48] [49]
Heparin	LC-MS, SAX ATR-FTIR	[50] [51]
Hyaluronic acid	HPLC-SEC Nanopore sensor	[52] [53]
Glucomannan	TLC, FT-IR, ^13^C NMR	[54]
*N*-Glycan	CE, MALDI-MS, ESI-MS	[55]

In general, deproteinization and extraction using water and organic solvents at low or high temperatures are frequently employed as pretreatments for collecting polysaccharides from natural resources prior to spectroscopic studies [56]. In addition, enzymes are used to cleave the covalent bonds in glycol polymers to separate monolithic functional carbohydrates from complex polymerized materials [57]. Those procedures are necessary for isolating polysaccharides from other ingredients such as proteins and analyzing them, but the stepwise routine process is often time-consuming.

Herein, a unique spectroscopic approach using a high-energy IR laser for the direct analysis of solid polysaccharides is presented. This method allows for the rapid and simple identification of chemical compounds in biological polymers by combining IR microscopy and ESI-MS [58,59,60].

The use of an intense IR laser has several advantages over conventional methods:

1. Conventional methods for biopolymer analysis include multistep procedures. The use of an IR laser can reduce the time required to determine rigid polymer structures (simplicity).
2. Conventional methods often use external organic solvents and extremely high temperatures to dissolve the rigid structure of polymers. However, IR lasers can perform direct analysis without any external treatments (simplicity and negative emissions).
3. The degradation process is completed within several microseconds because the laser macropulse is bunched with several hundreds or thousands of picosecond pulses (rapidity).
4. The IR laser can dissociate specific chemical bonds by tuning the resonant wavelengths in the target molecules (selectivity).
5. This effect is not caused by simple heating. Although general heating provides thermal energy to the entire compound, the excitation energy is deposited only on the corresponding chemical bonds.

## 2. Features of Method

Many biological and organic molecules possess mid-and near-IR absorption bands from 1000 to 4000 cm^−1^ (=2.5 to 10 μm): C=O stretching at 1600−1800 cm^−1^ (5.5−6.0 μm), N−H bending at 1400−1600 cm^−1^ (6.0−7.0 μm), H−C−O bending at 1200−1300 cm^−1^ (7.0−7.5 μm), and O−H, N–H, and C–H stretching vibrational modes at 2500−3000 cm^−1^ (3.0−4.0 μm) [61,62]. By irradiating an organic molecule with an IR laser, the IR multiphoton dissociation (IRMPD) reaction is induced by the vibrational excitation (VE) energy with the corresponding resonant wavelength, and the chemical bond is dissociated by the VE energy that exceeds the bond energy [63,64,65]. IRMPD can be facilitated using an IR free-electron laser (FEL) [66,67].

**Figure 1:**
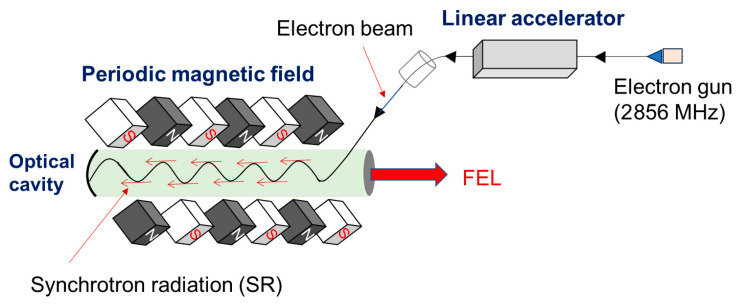
Oscillation system of free-electron laser (FEL). The system is composed of three major parts: linear accelerator, periodic magnetic field, and optical cavity.

FEL oscillation is based on the successive interaction of an accelerated electron beam (EB) with synchrotron radiation (SR) in a periodic magnetic field called an undulator (Figure 1) [68,69,70,71,72,73,74]. The EB originates from a radiofrequency (RF) electron gun (2856 MHz) and is accelerated to 100 MeV using an RF linear accelerator. The SR is stored in an optical cavity with concave mirrors at both ends and amplified through FEL interactions with the EB. Consequently, FEL lasing and power saturation are achieved, and highly coherent, intense laser light can be extracted through a coupling hole in one of the concave mirrors (Figure 1).

**Figure 2:**
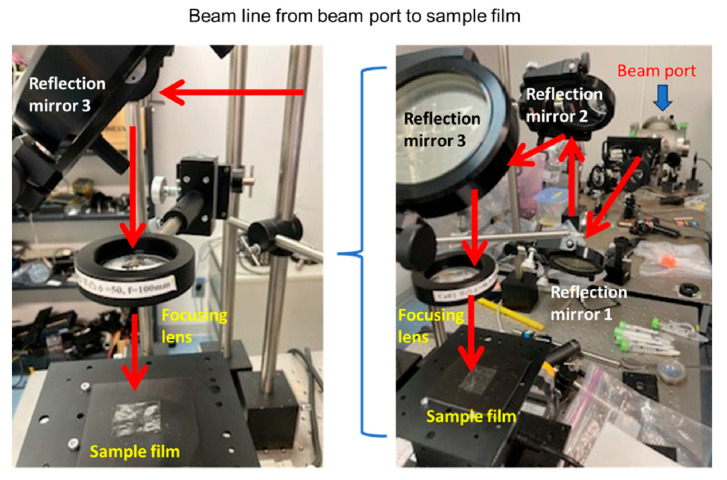
Example of irradiation setup. (**Right**): overall beamline. (**Left**): local beamline above sample film. The FEL is introduced onto the sample through three reflection mirrors and one focusing lens from the beam port. The photograph was taken at LEBRA, Nihon University.

The samples are typically placed on a slide base or in a glass tube, and the laser beam can be transported from the beam port onto the sample surface, as shown in Figure 2. Because the transported FEL beam is large (>10 mm in diameter), a focusing lens or off-axis parabolic mirror is used to focus the FEL beam onto the sample.

**Figure 3:**
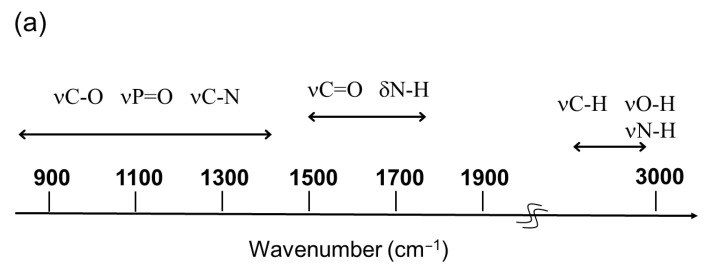

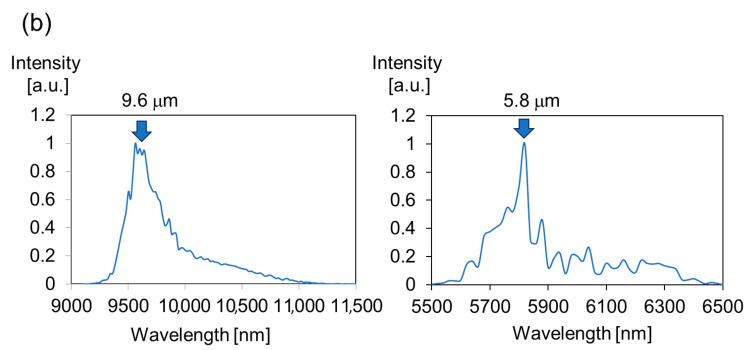
Characteristics of FEL. (**a**) Tunable wavelengths in IR region containing various vibrational modes of molecules. (**b**) Representative FEL spectra from 5.5 to 11.5 μm [75].

The IR FEL has unique characteristics compared with the thermal light source in a laboratory-level IR spectrometer, as shown in Figure 3.

(1) Tunable wavelength

The oscillation wavelengths of the FEL are generally tunable within the 2.0–20 μm (5000–500 cm^–1^) range, which covers the absorption frequencies of various vibrational modes of biomolecules (Figure 3a). Many organic molecules have a variety of vibrational modes, such as C=O stretching, N–H bending, H–C–O bending, and O–H stretching, at 1000–3000 wavenumbers (cm^–1^). FELs can excite these vibrational modes at their resonant wavelengths, and IRMPD can modify the chemical structures of many molecules in biological matter, gas-phase chemicals, and organic materials. The wavelength of the oscillation beam can be tuned by adjusting the space interval of the undulator or changing the kinetic energy of the EB. The typical FEL spectra at 5.8 μm (1724 cm^–1^) and 9.6 μm (1041 cm^–1^) are shown in Figure 3b [75]. The full width at half maximum (FWHM) was approximately 100–300 nm at both wavelengths.

(2) Pulse structure

The FEL has a complex temporal structure called “pulse train” or “burst pulse,” where several thousands of micropulses having 0.1–2 ps duration are bunched in one macropulse [70,71]. One macropulse had a 2–15 μs duration, and the repetition rate ranged from 2 to 5 Hz. The micropulse interval was 350 ps, which originated from the frequency of the electronic gun (2856 MHz). In the burst-mode experiments, the micropulse rate was divided by 64 (44.6 MHz), where one macropulse consisted of several hundred micropulses [72,73,74].

(3) High laser energies

The macropulse energy ranged from 5 to 25 mJ, and the beam diameter is generally set to ~200−400 μm on the sample surface using a parabolic mirror or a focusing lens made of CaF_2_ (f = 100 mm). The irradiation effect of the FEL on the sample at various fluences was investigated by varying the laser energy.

We applied the FEL to analyze solid-phase carbohydrate polymers using a combination of synchrotron radiation infrared microscopy (SR-IRM) and ESI-MS. The FEL can reveal the persistent structure of carbohydrates and release their monomeric units under atmospheric conditions, without any pretreatments.

SR-IRM has several advantages over thermal radiation beams in laboratory-level instruments: spatial resolution with a high signal-to-noise (S/N) ratio on a small area (several micrometers squares) of a small amount (several milligrams) of dry sample film [76,77]. An advantage of IR microscopy is that the position of the measurement region after FEL irradiation can be observed under an objective lens and can be determined by moving the sample stage automatically. The measurement can be performed in reflection mode, with a resolution of ~0.5 cm^−1^. Nonetheless, IR spectra are influenced by the surface shape of the sample film, and a limitation of the current measurement grade is that the image resolution is greater than several micrometers.

High-energy IR FELs can degrade various types of biopolymers such as peptide aggregates [78], melanin pigments [75,79], hydroxyapatite [75], cellulose [59], and lignin [60] by tuning the irradiation wavelengths resonant to each target compound in their solid states. This means that FELs are versatile and simple in analyzing biomacromolecules directly. The wavelength tunability implies, for example, that proteins are selectively degraded in the presence of nucleic acids by the FEL tuned to amide I (C=O stretching, 6.1–6.2 μm) because amide I is not included in the phosphate ester linkages in nucleic acids, but in the protein backbone. Furthermore, monomer units formed by more than C6 units are obtained from the parent polymers while maintaining an intact conformation. This is termed “functional degradation,” which is distinct from microbial degradation, in which the monomeric form is completely metabolized to C1-C3 units.

Analytical studies of two representative carbohydrates using FELs are shown as described below.

## 3. Analysis of Chitin

**Figure 4:**
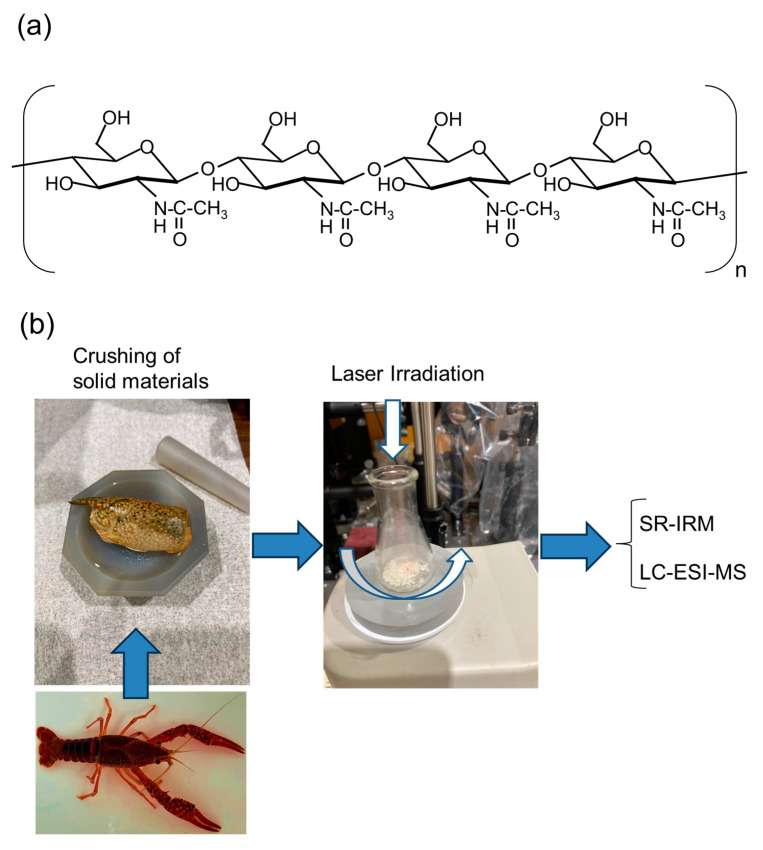
Analysis of chitin. (**a**) Chemical structure of chitin. n: polymerization degree. (**b**) Scheme of sample preparation and structural analysis of solid-state crayfish shells [58].

**Figure 5:**
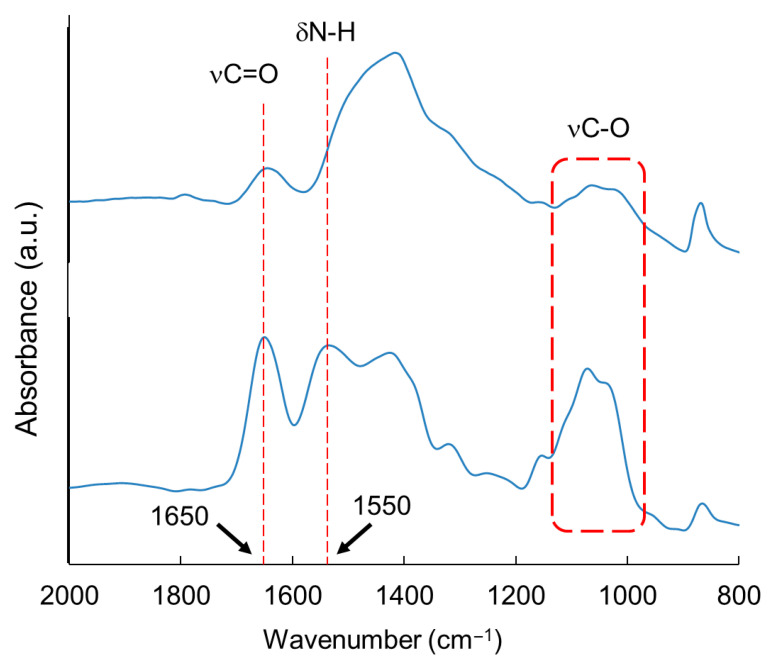
SR-IRM spectra of outer shell of crayfish [58]. (**Upper**): irradiation at 9.8 μm; (**bottom**): non-irradiation.

**Figure 6:**
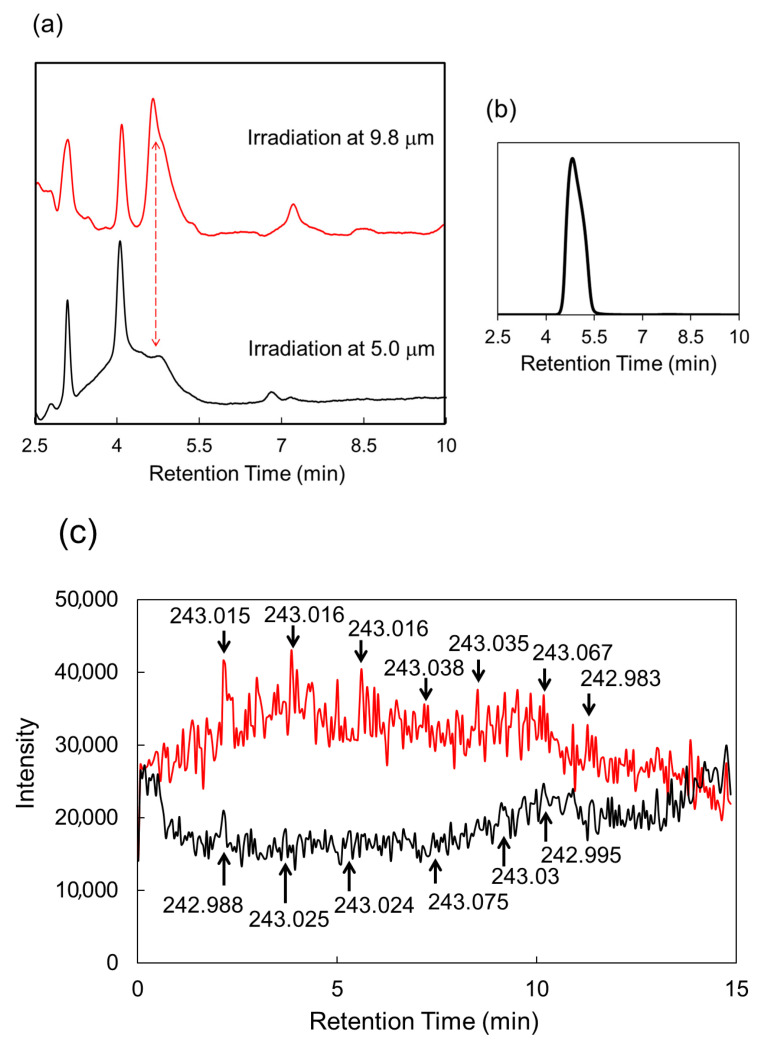
LC-ESI-MS analysis of crayfish arm after irradiation by FEL [58]. (**a**) LC profiles after FEL irradiation at 9.8 μm (red) and 5.0 μm (black). (**b**) LC profile of *N*-acetylglucosamine alone. (**c**) ESI-MS chromatograms of 243 Da after irradiation at 9.8 μm (red) and 5.0 μm (black).

Chitin is the main component of the outer shell of crustaceans, including shellfish, shrimp, crabs, and beetle [80,81] and is constructed mostly by *N*-acetylglucosamine (Figure 4a). We analyzed the natural powder from the outer shells of crayfish using an FEL oscillation system (Figure 4, Figure 5 and Figure 6) [58]. Several adult crayfish (*Procambarus clarkii*) were collected from local ponds and maintained in separate plastic containers under a dark–light cycle of 12 h/12 h, with fresh water changes during feeding. Five molting shells were collected, one solid arm was ripped from the shell, mashed in a mortar, and the resulting powder (ca. 50 mg) was placed in a 5 mL triangular flask. The powder was then directly irradiated by the FEL (Figure 4b). The SR-IRM in BL6B of UVSOR was used to detect conformational changes in the polysaccharide structure [82]. The instrument was composed of an IRT-7000 IR microscope combined with an FT/IR-6100 series spectrometer, and the mid-IR spectra were measured using a Michelson-type interferometer in reflection mode. The sample powder (~10 mg) was suspended in water (1 mL), and the mixture (20 μL) was placed on a stainless-steel base. After drying, the plate was set on a horizontally mutable stage and observed through a 16 × Cassegrain lens with an aperture of 50 μm × 50 μm.

The broadband peak at ~1100 cm^–1^ corresponds to the glycosidic bond (νC–O), and the intensity significantly decreased after irradiation at 9.8 μm compared with that of the non-irradiated sample (Figure 5). In addition, the peaks at 1550 cm^–1^ and 1650 cm^–1^ clearly decreased upon sample irradiation. This region contains the N–H bending and C=O stretching vibrational modes of *N*-acetylglucosamine residues in chitin. This implies that art of the carbohydrate chain in crayfish was cleaved, and *N*-acetylglucosamine was released by irradiation. Natural powder from crayfish contains several ingredients, such as calcium carbonate and proteins. However, a large portion of the crayfish arm is composed of chitin. In addition, the resonant wavelengths of the impurities are different from those of chitin, and the laser irradiation of the glycosidic bonds is specific to chitin. Therefore, these impurities do not interfere with the analytical results.

The LC-ESI-MS analysis results are shown in Figure 6. The elution peak at 4.6 min increased more in the case of irradiation at 9.8 μm than in the case of irradiation at 5.0 μm (low-absorption wavelength), while the other peaks hardly changed. The increased eluate peak indicates a higher amount of *N*-acetylglucosamine (Figure 6b). The mass chromatograms showed that the intensity of the mass peaks corresponding to 243 Da increased more at 9.8 μm than at 5.0 μm (Figure 6c). A mass value of 243 Da corresponds to a sodium-ion adduct of *N*-acetylglucosamine residue (221 Da) based on negative-ion-mode measurements (see Figure S1 for the standard sample).

**Figure 7:**
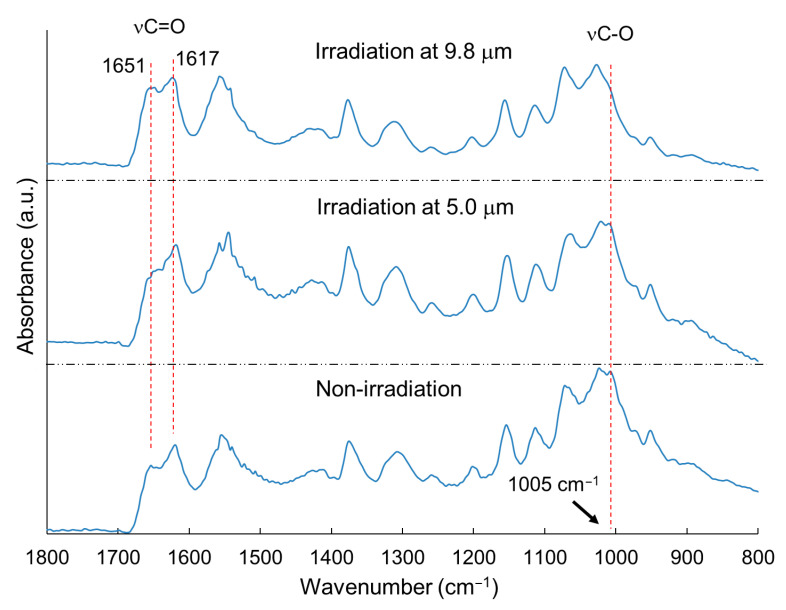
SR-IRM spectra of chitin powder [58]. **Upper**: irradiation at 9.8 μm; **middle**: irradiation at 5.0 μm; **bottom**: non-irradiation.

Commercially available chitin powder (~100 μm in particle size) was irradiated by the FEL, and the SR-IRM spectra of chitin are shown in Figure 7. The peak at 1005 cm^–1^, corresponding to νC–O, was decreased (upper) compared with the peaks resulting from irradiation at 5.0 μm (2000 cm^–1^) (middle) and non-irradiation (bottom) after irradiation at 9.8 μm (1020.4 cm^–1^). The amide carbonyl band at 1651 cm^−1^ (νC=O) in the spectrum of the *N*-acetylglucosamine residues was higher than the peak at 1617 cm^–1^ after irradiation at 9.8 μm (upper) compared with irradiation at 5.0 μm (middle). Therefore, the glycosidic bonds were cleaved, and the main chain of chitin was deformed by C–O-targeting irradiation, similar to the outer shell of crayfish. Taken together, FEL irradiation at 9.8 μm can be concluded to degrade the chitin chain and release *N*-acetylglucosamine units. This method requires no pretreatment such as boiling in acidic water or solubilization using organic solvents.

## 4. Analysis of Cellulose

**Figure 8:**
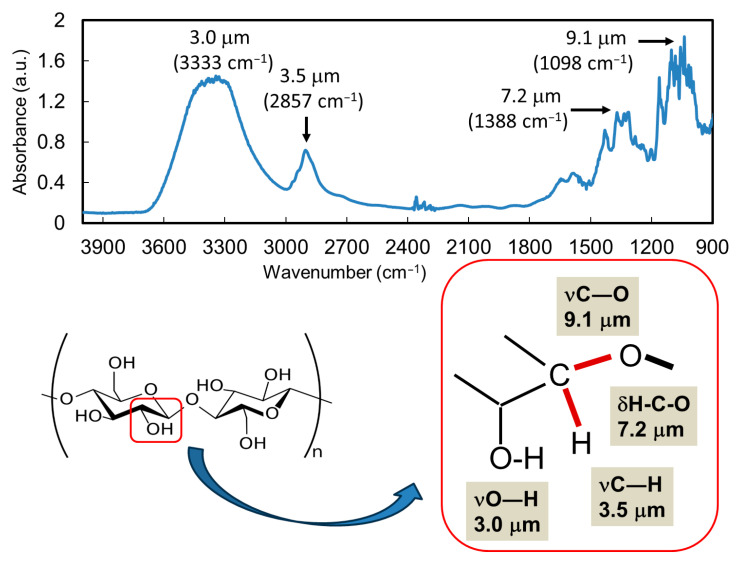
Wavelengths of FEL targeting glycosidic bonds in cellulose [59]. Upper: FT-IR spectrum; Bottom: resonant wavelengths around glycosidic bond irradiated by the FEL.

Cellulose is a major component of wood, and its degradation products, mono- and oligosaccharides, have attracted attention as carbon sources for bacteria fermenting bioethanol [83,84]. In addition, cellulose nanofibers have been developed as functional biomaterials, such as biocompatible cell membranes, antibacterial sheets, and food packaging composites, in the healthcare and pharmaceutical industries [85,86]. However, cellulose contains many glycosidic linkages and is generally difficult to chemically regulate. As shown in the SR-IRM spectrum of cellulose (Figure 8), four bands appear at 9.1, 7.2, 3.5 μm, and 3.0 μm [59]. These bands can be assigned to νC−O, δH−C−O, νC−H, and νO−H around the acetal carbon in cellulose, respectively. The FEL was tuned to these wavelengths, and the cellulose fiber was irradiated in a glass bottle at room temperature under atmospheric conditions. Commercially available cellulose little contains lignin because it is excluded during solid–liquid separation in the pulp-production process.

**Figure 9:**
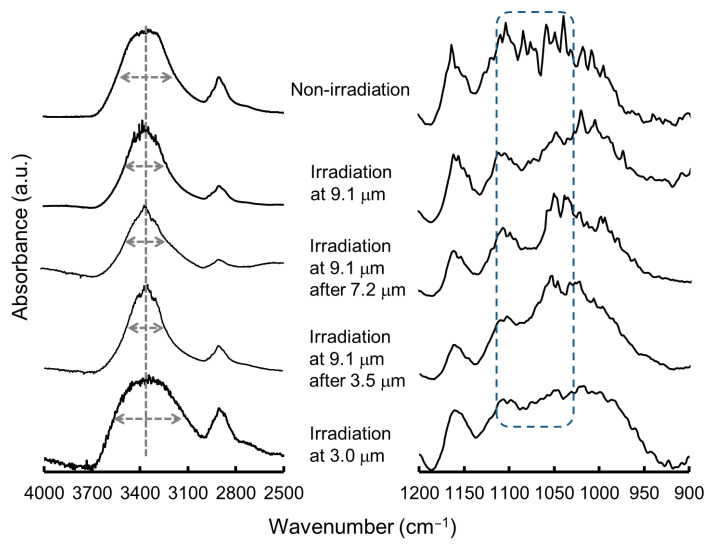
SR-IRM spectra of cellulose after FEL irradiation [59]. (**Right**): mid-IR region; (**left**): near-IR region.

Glycosidic bond cleavage was confirmed by SR-IRM analysis (Figure 9). The νC−O modes are apparent from 1000 to 1100 cm^−1^ (right panel), and after irradiations at 9.1 μm and 9.1 μm following 7.2 μm or 3.5 μm, these bands were clearly less intense than those arising from non-irradiation (top) and irradiation at 3.0 μm (bottom). In the NIR region at ~3400 cm^−1^ (gray dotted line), the half width (indicated by a double-headed arrow) was ~350 cm^−1^ in the non-irradiated sample (top) and 400 cm^−1^ in the sample after irradiation at 3.0 μm (bottom). On the contrary, all three irradiations (9.1 μm, 9.1 μm following 7.2 μm, and 9.1 μm following 3.5 μm) shortened the half width to ~300 cm^−1^. These changes in the hydroxy group region indicate that FEL irradiation, except at 3.0 μm, significantly altered the structure of cellulose from the non-irradiated sample. Combined with the spectral change in the mid-IR region, the results indicate that irradiation targeting the acetal group likely caused dissociation of the glycosidic bonds in cellulose.

**Figure 10:**
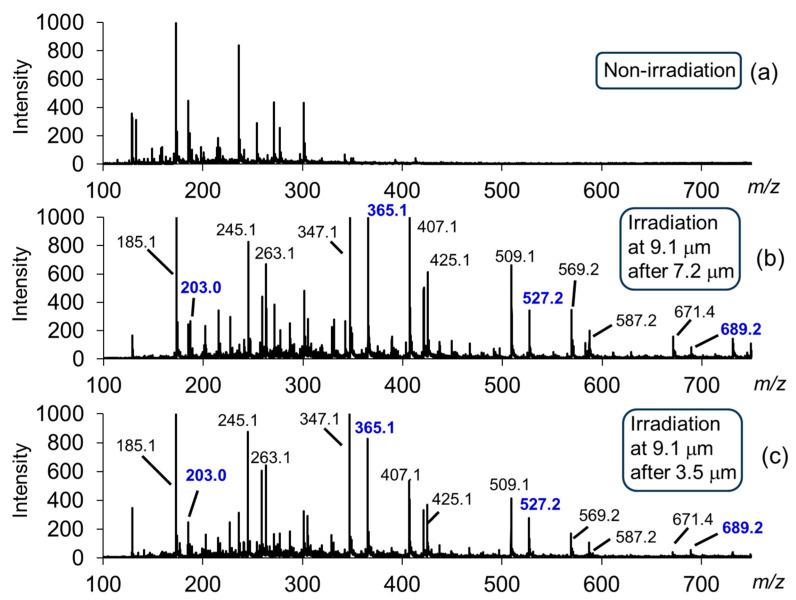
ESI-MS profiles [59]. (**a**) Non-irradiation; (**b**) irradiations at 9.1 μm after 7.2 μm; (**c**) irradiations at 9.1 μm after 3.5 μm. Blue numbers indicate the mono-, di-, tri-, and tetrasaccharides of glucose.

Figure 10 shows ESI-MS profiles of the non-irradiated sample (Figure 10a) and samples after irradiation at 9.1 μm following 7.2 μm (Figure 10b) and 3.5 μm (Figure 10c). Many more peaks were detected after irradiation compared with the non-irradiated samples. This indicates that these irradiations caused fragmentation of the cellulose chains. Mass peaks at 689.2, 527.2, 365.1, and 203.0 Da were assigned to tetrasaccharides, trisaccharides, cellobiose, and glucose, respectively, as sodium-ion adducts (see Figures S2 and S3 for standard samples of cellobiose and glucose, respectively).

**Figure 11:**
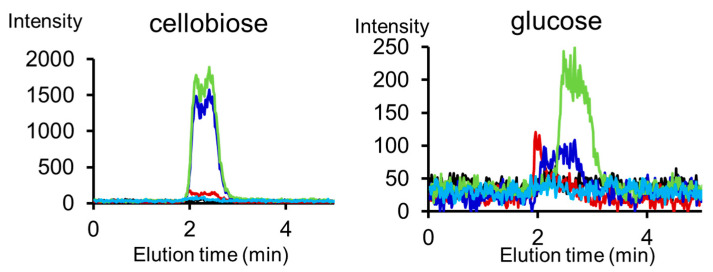
MS chromatograms of cellobiose and glucose after irradiation by FELs [59]. Black: non-irradiation; brown: irradiation at 9.1 μm; light green: irradiation at 9.1 μm following 3.5 μm; deep blue: irradiation at 9.1 μm following 7.2 μm; light blue: irradiation at 3.0 μm.

The production of cellobiose and glucose was also investigated using mass chromatography (Figure 11). The continuous irradiations at 9.1 μm following 3.5 μm (light green) and 7.2 μm (deep blue), afforded a greater amount of cellobiose than single irradiation at 9.1 μm (brown), and continuous irradiations following 3.5 μm was the most effective for glucose production. The VE at 3.5 μm can disrupt the interchain hydrogen bonds and unveil the fiber structure, which leads to cleavage of the glycosidic bonds by the IRMPD reaction at the C−O stretching vibrational mode. Intermolecular hydrogen bonds are formed between H2 and O6 in the cellulose structure [87]. A theoretical study can prove the destruction of the hydrogen bond network by the vibrational excitation of the H–C–O bending mode, which means that high energy can be deposited in the acetal group and affect the hydrogen bonds. Although no direct evidence for hydrogen bond destruction after 3.5 μm irradiation was found, the excitation energy of the C–H bonds in the acetal group can be considered to disrupt the corresponding hydrogen bond network in the cellulose.

Irradiation at 3.0 μm (light blue), which corresponds to the O−H stretching mode, did not produce saccharides. This implies that the glycosidic bond was unaffected by the activation of the hydroxyl group.

## 5. Discussion

In this study, we present a physicochemical approach using an intense IR laser as a “molecular scalpel” to analyze the solid-phase carbohydrate polymers chitin and cellulose as model samples. In chitin, the glycosidic bonds were cleaved by laser irradiation targeting the C−O stretching vibrational mode (9.8 μm), and *N*-acetylglucosamine residue was released from the crude shells of crayfish. In the case of cellulose, continuous irradiation targeting the C–O and C–H stretching modes was effective for cleaving the glycosidic bonds. The VE supplied to the acetal group can be considered effective in degrading polysaccharides into their monomeric sugars.

The yield of sugars from the carbohydrate polymers was several percent of the total eluate, as determined by chromatographic analysis. The efficiency of bond cleavage depends on the laser energy. An energy of ~10–20 mJ per macropulse is required to obtain this amount of monomeric product from the polymerized structure, and an energy below 1 mJ is ineffective. The reproducibility is good as long as high-energy laser oscillations are continuously maintained during the experiment. The sensitivity of detection depends on the sample preparation and measurement instruments, and several milligrams of the sample powder can be analyzed using SR-IRM and ESI-MS. In addition, the detection limits are proportional to the laser energy of the FEL. When the laser energy is low (below 1 mJ), the conformational changes in the samples cannot easily be observed microscopically, and fragmentation of the compounds is difficult to detect using MS.

Mechanistic insights can be considered as follows:

In the process of C–O bond dissociation, the IR multiphoton excitation reaction generates vibrationally hot molecules, and radical cation species can be produced [67]. This reaction intermediate can be rapidly converted into thermostable products such as glucose. An FEL is essentially a picosecond-pulse laser, and the dissociation reaction can occur within several hundred picoseconds. In fact, one or two macropulse irradiation is sufficient to disperse the target structure of rigid biopolymers, as shown by the dissociation of melanin by the FEL [79]. The aromatic compounds can be degraded by several shots of irradiation to release some of the pyrrole groups.

Glycosidic bonds can potentially be cleaved by thermal effects. In general, acidic solutions (pH 2–4) induce acetal bond cleavage at high temperatures. However, FEL irradiation is performed without such acidic solvents; thus, this possibility can be excluded as long as the present irradiation conditions are met.

In addition, general heating provides thermal energy to the entire structure of molecules, but VE provides energy only to the corresponding chemical bonds in the molecule.

The physical method of using an intense IR laser requires no specific conditions such as acidic or alkaline solutions, organic solvents, high pressures, or high temperatures, although high electric power is needed to maintain instrument operation. A benefit of using FELs for the analysis of complex polymers is their wavelength tunability. For example, both glycosidic bond cleavage and hydrogen bond dissociation in the assembled carbohydrate chains can proceed in a single sample tube by changing the irradiation wavelength. Furthermore, the laser-induced dissociation reaction can be completed within several shots of the macropulse (several milliseconds) owing to the pulse structure of the FEL. Compared with biochemical systems that use microbial enzymes, the rapidity and simplicity of the experimental setup is a major advantage. Using a similar approach, we can modify lignin, which is one of the major components of wood along with cellulose, and the rigid aromatic polymer can be analyzed using FEL irradiation [60].

To analyze complex ingredients in real-world samples, fossilized inks from cephalopods were used as a model [75]. This natural biomaterial contains melanin, proteins, hydroxyapatite, and inorganic salts. Melanin can be distinguished from hydroxyapatite by selecting the FEL wavelengths corresponding to the C=O bond in the indole and pyrrole groups of melanin. The structural differences in hydroxyapatite and melanin in GSM122841 collected from the Peter Borough Member of the Oxford Clay Formation and YPM221210 collected from the Koblenzer Bed of the Posidonia Shale Formation were determined. Therefore, the use of laser irradiation is robust and generalizable to natural complex samples in the solid state.

Finally, we propose other possible applications for FEL in polymer science. Similarly to natural carbohydrates, textile fibers are rigid polymers. For example, fiber plastics, such as polyester and polyamide, and fiber proteins, such as wool keratin and silk fibroin, are polymerized by covalent C–C, C–O, and C–N bonds [88,89,90,91]. Textiles often contain dyes that pollute the environment [92]. To analyze dyes, heating with strong acids or alkaline solutions is employed as a pretreatment [93]. Textile dyes were directly analyzed using matrix-assisted IR laser desorption electrospray ionization mass spectrometry [94]. In that study, the IR laser was tuned to 2.9 μm, corresponding to the O–H stretching mode of water, which induced the desorption of textile fiber particles. We propose the use of an FEL as a molecular dissection tool for analyzing textile fibers. Textile organic materials exhibit many IR absorption bands, such as C–O stretching and N–H bending modes, and irradiation experiments can be performed on these organic fibers if the FEL can be tuned to these wavelengths. The fragmentation patterns of the fibers are expected to differ depending on the irradiation wavelength, which allows us to estimate the molecular compositions of the fiber structures. In addition, monomer materials can be produced from rigid polymerized fibers using laser irradiation, and the system can be applied to regenerate textiles wasted in soil and ocean environments.

As another persistent polymer, perfluorinated and polyfluorinated alkyl substances (PFAS) and small amounts of contaminants, including those in drinking water, can be analyzed using solid-phase extraction followed by liquid chromatography/tandem mass spectrometry (LC/MS/MS) [95]. In addition, the PFAS content and volatile organic compounds in kitchenware have been analyzed using GC–MS [96]. We can assume that FEL irradiation would affect the conformation of PFAS depending on the specific wavelength. The stretching vibrational mode of C–F is observed at 1000–1500 cm^–1^, and the VE energy targeting the C–F bonds can be deposited onto the fluorinated molecules, which can induce degradation of the backbone conformation. This FEL pretreatment can be applied to subsequent analyses using conventional chromatography and MS. These studies are currently underway at our laboratory.

## 6. Conclusions

We briefly summarized the various analytical techniques for carbohydrate polymers and described an original spectroscopic approach employing an intense IR laser in polymer analysis. The FEL oscillates in the IR region from 1000 cm^–1^ (10 μm) to 4000 cm^–1^ (2.5 μm) and can be applied to irradiate rigid carbohydrates such as chitin and cellulose. After FEL irradiation of the solid-phase materials at resonant wavelengths under atmospheric conditions, each monomeric sugar and oligosaccharide was detected using ESI-MS, and the conformational changes in the parent polymeric structures were observed using SR-IRM. The fragmentation and degradation mechanisms are based on the IRMPD reaction, which is induced by VE energy from a linear accelerator. The IR laser described herein can be used not only to determine the structures of rigid carbohydrate polymers but also as a pretreatment method for dissolving intact polymerized structures before separating them from other ingredients using LC and analyzing them using MS. In addition, the FEL technique can be adopted to irradiate many types of biological and organic molecules by tuning the irradiation wavelengths to their specific vibrational modes. However, the current FEL system is installed at a synchrotron radiation facility, limiting its accessibility. A laboratory-level IR laser that possesses both high laser energy and wavelength tunability is expected to be developed in the future.

## Data Availability

The Original data are available upon request from the corresponding author.

## Supplementary Materials

The following supporting information can be downloaded at: https://www.mdpi.com/article/10.3390/polym17172273/s1, Figure S1: MS analysis of *N*-acetylglucosamine; Figure S2: MS profile of cellobiose; Figure S3: MS profile of glucose.

## Abbreviations

The following abbreviations are used in this manuscript:

FEL	Free-electron laser
IRMPD	Infrared multiphoton dissociation
SR-IRM	Synchrotron radiation infrared microscopy
ESI-MS	Electrospray ionization mass spectrometry
SR	Synchrotron radiation
VE	Vibrational excitation
EB	Electron beam
RF	Radiofrequency
HPLC	High-performance liquid chromatography
CE	Capillary electrophoresis
NIR	Near-infrared
AFM	Atomic force microscopy
SEC	Size exclusion chromatography
MALLS	Multiple-angle laser light scattering
NMR	Nuclear magnetic resonance
DSC	Differential scanning calorimetry
HPAEC-PAD	High-performance anion exchange chromatography with pulsed amperometry detection
MALDI-MSI	Matrix-assisted laser desorption/ionization–mass spectrometry imaging
PAS	Photoacoustic spectroscopy
TG	Thermogravimetry
XRD	X-ray diffraction
SEM	Scanning electron microscopy
SAX	Strong anion exchange
MS	Mass spectrometry
ATR	Attenuated total reflectance
LC-MS	Liquid chromatography mass spectrometry
TLC	Thin-layer chromatography
