# Supplementary material for: Direct Analysis of Solid-Phase Carbohydrate Polymers by Infrared Multiphoton Dissociation Reaction Combined with Synchrotron Radiation Infrared Microscopy and Electrospray Ionization Mass Spectrometry
Source: Polymers (Basel). 2025 Aug 22;17(17):2273. doi: 10.3390/polym17172273 (PMC12431057; doi:10.3390/polym17172273)
Supplement: Supplementary file 1 [file polymers-17-02273-s001.zip › polymers-3788917-supplementary.pdf]

Supporting Information

for

Direct Analysis of Solid-Phase Carbohydrate Polymers by Infrared Multiphoton Dissociation Reaction Combined with Synchrotron Radiation Infrared Microscopy and Electrospray Ionization Mass Spectrometry

Takayasu Kawasaki[1,*], Heishun Zen[2], Kyoko Nogami[3], Ken Hayakawa[3], Takeshi Sakai[3] and Yasushi Hayakawa[3]

[1]Accelerator Laboratory, High Energy Accelerator Research Organization, 1-1 Oho, Tsukuba 305-0801, Ibaraki, Japan.

[2]Institute of Advanced Energy, Kyoto University, Gokasho, Uji 611-0011, Kyoto, Japan.

[3]Laboratory for Electron Beam Research and Application (LEBRA), Institute of Quantum Science, Nihon University, 7-24-1 Narashinodai, Funabashi 274-8501, Chiba, Japan.

*Correspondence: takayasu.kawasaki@kek.jp

ESI-MS data of standard sugars.

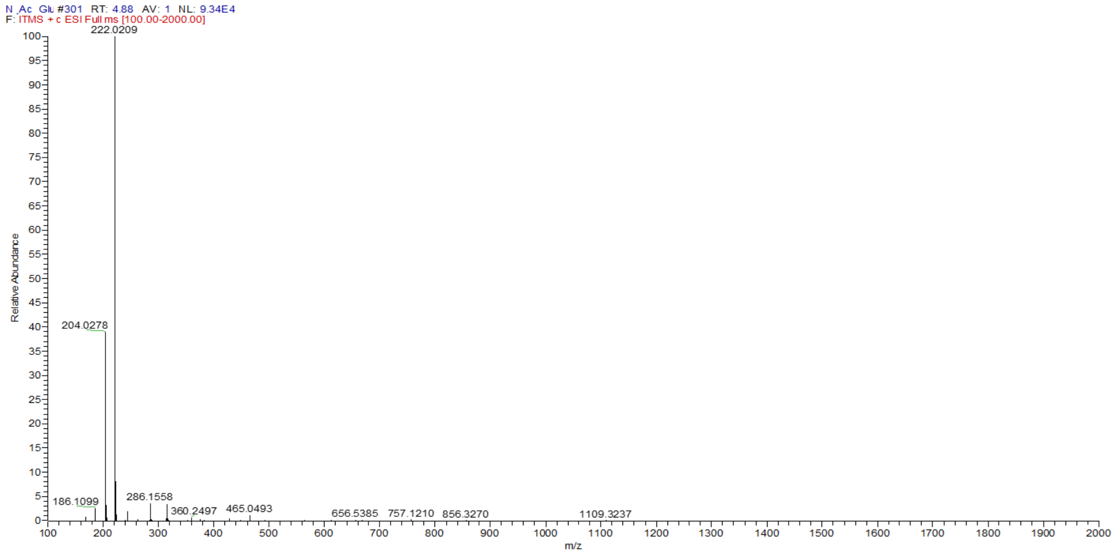

Fig. S1. MS profile of *N*-acetylglucosamine at positive ion mode. Measured mass: 222 Da; Theoretical value: 221 Da.

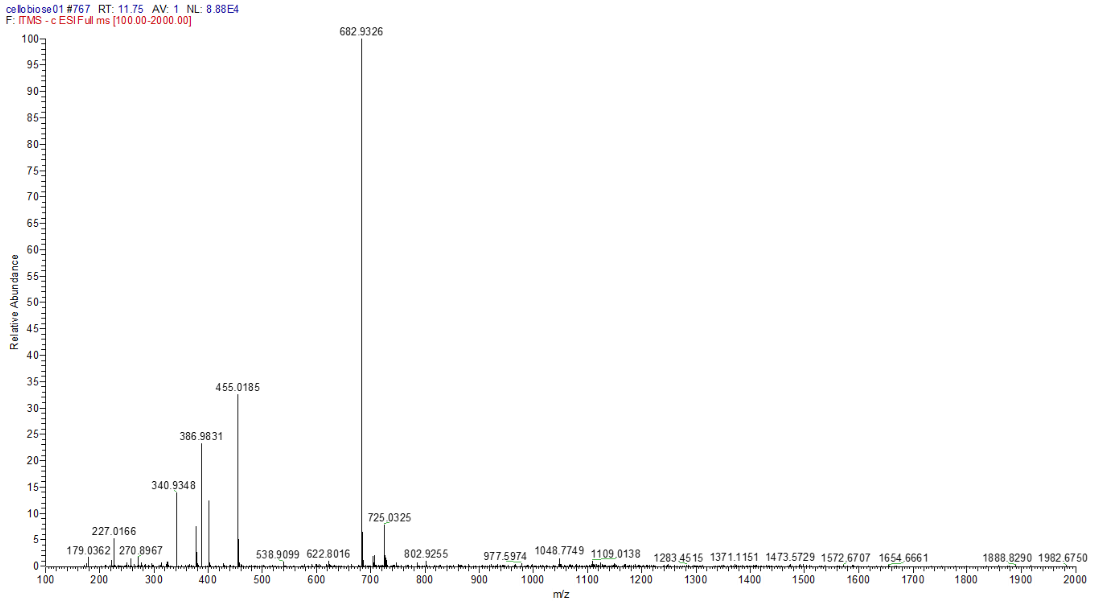

Fig. S2. MS profile of cellobiose at negative ion mode. Mesured MS: 341 (monomer), 683 (dimer); Theoretical value: 342 Da (monomer), 684 Da (dimer).

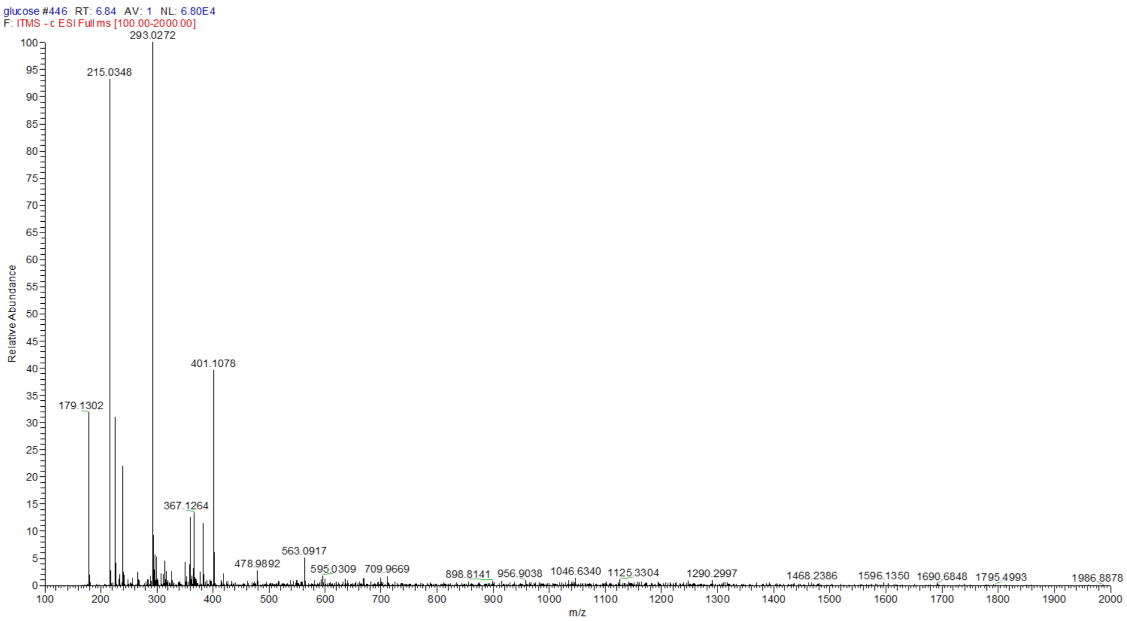

Fig. S3. MS profile of glucose at negative ion mode. Mesured MS: 179 Da; Theoretical value: 180 Da.
